# Diarrhœa

**Published:** 1888-12

**Authors:** 


**Diarrhœa.**—A combination of pepsin, subnit. of bismuth and Dover's powders has been especially successful in the treatment of infantile diarrhœa. While the use of salol is still in embryo, it apparently gives fair promise.—*Ind. Med. Jour.*

